# Observations on the Surgical Pathology and Treatment of Aneurism. Part 1

**Published:** 1841-10-01

**Authors:** 


**OBSERVATIONS ON THE SURGICAL PATHOLOGY AND TREATMENT OF ANEURISM, BEING THE SUBSTANCE OF A COURSE OF LECTURES ON THAT DISEASE. Delivered in the School of the Royal College of Surgeons in Ireland during the Session 1839—40. Part I. By *William Henry Porter*, A. M. &c. &c. Octavo, pp. 214. Dublin, J. Porter. 1841.**

MR. PORTER informs us that this book had its rise in the following manner:—

"A short time previous to the Winter session of 1839-40, some cases of aneurism occurred under my care in the Meath Hospital, attended with unusual symptoms, and followed by important, and, in some respects, unexpected results. It became, of course, my duty to endeavour to explain the nature of these cases to the pupils attending the institution; a task which I found to be attended with more difficulty than I could have anticipated, in consequence of the vague and indistinct ideas entertained by many of them as to the pathology and treatment of even the simpler and more ordinary forms of the disease. It seemed, indeed, to be the prevailing opinion that aneurism consisted in some particular lesion of an artery, to be cured, if cureable at all, by one particular operation, the only objection to such operation being the situation of the disease in a locality where the vessel leading to it could not be reached; but, with respect to the existence of different pathological forms of the disease, and of the applicability of a suitable line of treatment to each variety, I discovered a good deal of incertitude or misconception. This deficiency, I confess, surprised me, when I reflected on the zeal and ability with which this branch of surgery has been of late years cultivated: but I found there was still something wanted, some short yet comprehensive arrangement, founded solely on pathology, connecting each variety with the operation or other treatment adapted to it, and explaining (or at least endeavouring to explain) the circumstances that so frequently mar the best concerted operations and accelerate a patient's dissolution." vi.

And so these lectures originated. In the arrangement of his subject he has endeavoured to treat of aneurism in its generic forms, (as far as is practicable) independent of the proper and peculiar features that may attach to it in any particular locality—to describe the different pathological conditions that precede and are supposed to conduce to the formation of the disease—to connect these with the symptoms as they arise—and to point out the mode of treatment apparently suitable to each variety. Another object with him has been to endeavour to ascertain how far our curative measures may be insufficient, and our operations unsuccessful, and to explain (to the extent our pathological information will permit) the causes and circumstances that thus exert an unhappy influence on our practice.

The first subject on which our author treats is HÆMORRHAGE. We shall glance at any peculiar opinions he may entertain.

He seems to think the internal coagulum of little service. It never occupies, says he, the entire calibre of the vessel, and, consequently, cannot block it up mechanically; and as its presence or absence depends on the distance between the wound and the next branch, it should follow, if it is instrumental in controlling hæmorrhage, that an artery divided close to such branch would never cease to bleed. This is by no means found to be the case, and therefore are there grounds for believing that the existence of an internal coagulum is more or less accidental, and not in any respect necessary, or even important.

We do not quite agree with Mr. Porter upon this head. If the internal clot be unimportant, how comes it that hæmorrhage is much more *likely* to happen if it is not present. Most surgeons will admit the greater tendency, on the part of a vessel, to bleed, when injured near a collateral branch, than when away from it. That surely must depend in a material degree on the state of the internal clot.

Mr. Porter remarks, and justly enough, that lacerated arteries sometimes bleed furiously, the natural process of arresting hæmorrhage not being always efficacious. Not long since, a man was brought into the Meath Hospital, whose fore-finger was broken and lacerated in a steam-engine—he resisted amputation, and for two days this apparently trifling wound poured out blood with a violence absolutely incontrollable. At length he submitted to the operation as the only means of saving him from bleeding to death; and from the clean incised wound thus made, not a drop of blood flowed—neither was it necessary to tie a single ligature. Very recently, in the case of a man whose arm was torn off by a steam-engine, the main artery of the limb hanging from the wound bled with a degree of violence that was nearly fatal before he could be conveyed to hospital. It appears, then, that the means adopted by nature to control hæmorrhage are not the same in this case as when an artery is simply divided by a cutting instrument, and that they are not uniformly brought into operation; but as these means are not understood, neither is it possible to explain the causes of the different phenomena that occur under circumstances so apparently similar.

Mr. Porter inquires into the method by which a notched or punctured artery heals.

"Are the processes just described the same by which an artery that is only notched heals subsequently—or is it a matter of necessity that a vessel once opened in any manner must continue to bleed, unless its calibre is obliterated? On this subject I have met with a considerable diversity of opinion, and there has not been a sufficiency of evidence collected to decide the question satisfactorily. Many persons, for whose practical experience I entertain the highest respect, believe that an artery so circumstanced may heal without obliteration. Dr. Jones details three experiments, the results of which would show that the arteries of dogs, if only divided to the extent of one-third of their circumference, heal without their calibres being even diminished; but with reference to this point it must be remarked that the arteries of inferior animals do not, either in their physiological or pathological qualities, resemble those of man, and therefore, (to say the least of them,) all such experiments prove nothing in explanation of the process of repair in the human subject. Again, a case of aneurism, at the bend of the arm, and produced by the puncture of a lancet in bleeding, was treated by compression in Steven's Hospital, and recovered; the artery remaining perfectly pervious through the entire of its course; but in this case the vessel passed in front of the tumour, and the only way in which a connexion between them could be explained was by supposing the lancet to have transfixed the vessel, the anterior wound to have healed, and the posterior to have formed the aneurism. But this is stretching imagination beyond probability—and it is far more likely that the artery which passed in front of the tumour was the radial which came off, (as it frequently does), very high up, whilst the ulnar, occupying the usual situation of the main trunk, had been wounded, and the aneurism, in increasing in size, pushed forward the radial on its surface. Under all circumstances, however, and fortified by the uniformity and simplicity with which nature performs her operations, I freely acknowledge that I do not believe a wounded artery ever heals unless by obliteration. Already it has been shewn that in an open and patulous wound, a notched artery will bleed to a terrific extent, merely because it cannot retract, and a coagulum cannot be formed. I have known death to be the result of such a wound of the anastomotica magna, near the condyle at the elbow, in a case that was left without assistance." 19.

Mr. Porter passes from the physiology of hæmorrhage to the treatment of it. Speaking of compression, he says :—"a graduated compress is to be placed exactly over the aperture in the bleeding vessel, and secured there by a bandage, rolled so evenly, that no one part of the limb is subjected to greater pressure than another; and so firmly as to lay the opposite sides of the vessel fairly in contact, but, at the same time, occasion as little pain as possible. This is generally accomplished by a comparatively moderate degree of pressure, and I believe the important point to be attended to is the evenness of the bandage rather than the force it exerts. In the arm, for instance, which is frequently the seat of this operation, in consequence of the artery being punctured by an awkward and ignorant phlebotomist, the bandage should commence with the fingers, each of which should be rolled separately and firmly, it must then be carried evenly up the arm, and terminate a short distance above the position of the compress. The arm should then be laid upon a pillow, and the patient receive the strictest injunctions not to attempt to move it. For some hours the case will require the most watchful attention on the part of the surgeon. If there is no increase of pain, or throbbing, or other uneasiness, the compression will probably succeed, and I believe many an individual has had the brachial artery opened without ever entertaining a suspicion that he had been exposed to so much peril: if there is this increase of pain or tension, the arm must be opened and examined lest bleeding should be going on internally."

Mr. Porter, of course, enjoins our chief dependence on the ligature. And he gives a hint which might be taken on some occasions with advantage. In order, he remarks, to secure an artery that has been perfectly divided, each segment should be seized with a tenaculum or forceps, gently drawn out from the surrounding cellular tissue, and held whilst an assistant ties up the mouths of the vessel with a round ligature properly prepared. In every instance, with one exception, the vessel should be completely insulated, and nothing but itself included within the cord: that exception having reference to the arteries of old persons which have become rigid and contain a quantity of earthy deposit, and are apt to break off during the operation of seizing and drawing them out. This may render it excessively difficult to secure such a vessel, and, in such a case, it will be advisable to include some of the adjacent softer tissues, (always excepting a nerve or a vein, which, under every disadvantage, ought to be avoided,) and the patient thus treated, in general, progresses very favourably. From inattention to this precaution, Mr. Porter has more than once seen great difficulty and delay in securing the arteries of a stump after amputation, and the operator at length obliged to resort to deep plunges of the needle.

Mr. Porter enumerates the different varieties of aneurism, inserting amongst them one with which our readers may not be familiar.

"There is a pathological condition of an artery producing all the symptoms of circumscribed false aneurism, and apparently curable by the same means, which, as yet, has only been noticed and described by myself in the Cyclopædia of Anatomy. It seems to be formed by a dilatation of the fibrous and cellular coats, and the absorption of the internal lining. It appears to be so far a true aneurism as that the artery is uniformly dilated around its entire circumference, and it is so far a false one, that the internal lining membrane has been removed. I know not how to form a name for such a complex aneurism." 34.

Mr. Porter devotes some little attention to the causes of aneurism. He adverts to certain facts that he has made out.

1. It is a disease unknown to early life, not, (as far as I know,) having been observed antecedent to the age of puberty, and although old age is not absolutely and completely free, it is comparatively rare at that period; from an aggregate of thirty remarkable cases operated on, I find the average to be thirty-three years and a half.

2. It is much more frequently met with in males than in females: this is remarkably proved in cases of internal aneurism, but still more so in the external. I have made the most extensive inquiries on this subject in my power, and can find but one surgeon in Dublin who ever operated for the cure of popliteal aneurism in the female.

3. Arteries are liable to aneurism with a frequency nearly in proportion to their size: thus the most common seat of all is the aorta: and, again, particular parts of these are more liable than others, aneurisms usually having their seats where the arteries are curved or bent, or where a branch may happen to have been given off.

Mr. Porter adds:—

"Seeing, then, that aneurism is at least negatively subject to certain laws—that it is not met with in animals—that it is not met with in the human subject before the age of puberty, and rarely in the female at any age—and that it prevails in the larger arteries, we must seek some explanation of its existing cause that will be consistent with these facts. But first it may be necessary to make a few remarks on the causes usually assigned. It is said that certain laborious trades and occupations predispose to aneurism, but this opinion cannot be supported. If it be meant that such persons are more exposed to injury, and that direct violence may sometimes rupture an artery, this is as easily understood as that a person working in the neighbourhood of a steam-engine will be more likely to be entangled therein, and have a limb torn off, than a person at a distance from it; but this is only accidental, and by no means a particular liability to disease. I cannot believe that any degree of labour, or exertion, or exposure, predisposes to the occurrence of aneurism; first, because the most laborious people in society, sailors, blacksmiths, porters, &c., are not a whit more liable to the disease than any other classes in the community: secondly, because women in the lower ranks of life are, in many instances, obliged to undergo more labour in proportion to their strength, than men—and boys and children infinitely more so; yet we have seen the exemption that these classes enjoy: and, thirdly, aneurism is not more prevalent among the poor and laborious than among the rich and idle, in proportion to the relative numbers of these classes respectively. This latter assertion is so opposite to the generally received opinion, that many will be inclined to disbelieve it, and its truth cannot be proved for want of statistic reports; I have made it, however, from my own observation, and would appeal to any practical surgeon of experience for its corroboration. Again, it has been stated that particular trades and callings, chiefly such as oblige their followers to keep their limbs in a bent posture, predispose to aneurism, and this is explained by the idea of the blood being forcibly impelled against the side of the vessel, in consequence of such position. Hence the prevalence of the disease amongst coachmen, postilions, horse-soldiers, &c. But there can be no doubt that other persons are equally, if not more, exposed to such influences, in whom the disease does not prevail—studious persons, and the higher orders of females, for instance: but with reference to trades, we may recollect that tailors spend most of their time in a very contracted position, and yet no one ever considered them as peculiarly predisposed to aneurism. I cannot, therefore, admit any of these as creating a predisposition to the disease." 39.

We confess that we are startled by this reasoning of Mr. Porter's. So far as we have seen, we should say that aneurism is more frequent amongst the laborious than the indolent classes of society. And so, we believe, are those alterations in the coats of arteries that lead to aneurism. This seems to us a matter of fact. As a matter of reasoning, it is consistent with all physiological probability that diseases of the arterial system should be most rife in those whose vessels are exposed to most wear and tear. The argument deduced from the infrequency of aneurism in women and in children appears to tell rather against Mr. Porter than for him. Women certainly do not make such violent exertions as men, nor do children. And as such exertions tell rather by their cumulative than direct effect, we should scarcely expect aneurism in the hard worked boy, though we might fairly look for it when that boy has become the broken down man.

Mr. Porter thinks that the condition of the arterial tunics which induces aneurism, or disposes to it, is unhealthy inflammation of the tunics. By this their qualities of elasticity and contractility are greatly diminished, and they will yield and become distended under the ordinary force of the circulation; hence we may understand how some of the pathological conditions of the artery come to be produced. First, the vessel may be rendered distensible throughout its entire circumference and thence dilated all round, in such a manner as to have acquired the name of *fusiform* dilatation. An artery, however, in this state cannot be considered as aneurismal: the blood circulates through it during life, and no portion of it is coagulated or withdrawn from the system. After death, indeed, a coagulum is always found within it—an additional proof, if such was wanted, that its contractility was impaired and weakened. But one spot of this fusiform dilatation may be weaker than the rest; and if so, it will be likely to yield, become dilated, and a true aneurism thus be formed, in which blood will be arrested and coagulated during life.

Mr. Porter, then, is satisfied that unhealthy inflammation is one of the most influential of the exciting causes of aneurism, and he has been led to the conclusion that intemperance, particularly in the abuse of ardent spirits, and repeated or ill-conducted courses of mercury have, at least, some intimate connexion with it. This latter medicine is seldom used in great quantities, or for any protracted length of time, unless for the cure of syphilis, and hence many have entertained an opinion that arteritis might be a result of the operation of the venereal poison. We are inclined to imagine that intemperance and abuse of mercury predispose to aneurism as to many other diseases, by impairing the general constitutional powers. Local or accidental circumstances determine the particular malady that shall ensue.

Mr. Porter passes to *Internal Aneurism,* the symptoms and diagnosis of which he first considers. He offers some suggestions on the latter head which we may notice. A gland, he says, or other tumour, receiving an impulse from an artery, is merely lifted up, and the pulsation is only felt at the apex—often by drawing it away from the vessel the pulsation ceases altogether: an abscess receives only an undulatory thrill from an artery, perceptible in the line of the vessel, but fading away and becoming indistinct in the remoter parts of the tumour: but the expansion of an aneurismal sac is equal in every part and every direction, and the pulsation can be felt as correctly at the base or at the side, as at the summit. Occasionally some tumours of a medullary or other fungoid nature exhibit a pulsatile character, and, if they occupy a situation in the course of a large artery, are liable to be mistaken for aneurism—indeed the resemblance is sometimes so great as to render a diagnosis extremely difficult. Nor will the position, growth, or direction of the tumour be sufficient to determine the point in question, for we know that aneurisms are uncertain in these respects, and may present themselves in very unusual and unexpected situations. He has known a popliteal aneurism, for instance, to appear on the front of the leg, beneath the knee, and it was curious that, at the same time, he had a patient in hospital with a fungoid tumour in exactly the same situation that exhibited a decidedly pulsatile character. He thinks the pulsation of a fungoid tumour is less forcible, less distinct, less *prononcèe,* (as the French would term it,) than that of aneurism. But he has heard of cases in which the diagnostic symptoms were so obscure as to occasion considerable embarrassment to very acute and sagacious practitioners. Hence the necessity of making the most rigid investigation of every particular connected with a tumour of suspicious nature, for we shall find hereafter that aneurism is a disease in which even a trifling error may give rise to formidable, if not fatal, consequences. On applying the ear or a stethoscope to an aneurismal tumour, a peculiar sound, termed by the French the *bruit de soufflet,* is often, indeed generally, heard. It conveys the impression as if a rush of air took place into the sac at each pulsation; but as it is impossible to convey any idea of it by description, the practitioner must seek to render himself familiar with it: still, however, it is not pathognomonic, for the fungoid, or indeed any tumour situated on an artery will produce it, and it may be created by artificial pressure. It is almost needless to add that the mistake of confounding aneurism with fungoid tumour has been committed by the best surgeons, and is too easily made.

Mr. Porter runs over the sites of internal aneurism. He has seen one within the cranium. It was about the size of a small bean, in the basilar artery, the coats of which contained the same kind of earthy deposition that pervaded all the other arteries in the body, and which, therefore, must have been formed by dilatation only. The subject had been a man of some consequence, who suffered greatly from, and eventually died of, urinary disease. During life he exhibited some derangement of the cerebral functions, which was attributed to sympathy with the bladder—he became absent, totally forgetful, listless, sleepy, and almost comatose; finally he died, and this little aneurism was found, the rest of the contents of the skull being perfectly normal and healthy.

Speaking of thoracic aneurism Mr. Porter observes, what we fear is too true, that there cannot be a doubt that many cases have occurred, in which "the stethoscope discovered no sign of disease in the heart or aorta," nor can this be attributed to careless examination, or want of competence in the auscultator; for no later than the past winter, a specimen of aortic aneurism was *exhibited,* which, during life, had been mistaken by more than one, and treated as pulmonary consumption. Yet it often affords valuable information even in cases of this kind. Mr. Porter adduces the following as symptoms of thoracic aneurism. There is generally some irregular action of the heart—irregular is the only word that can be used; for, in this respect, there are almost endless varieties—sometimes it is wonderfully increased—there are palpitations, faintings, and other symptoms usually considered as indicative of the existence of organic disease in this viscus: sometimes the heart's action is regular on ordinary occasions, but becomes strongly excited by walking up stairs, or any similar exertion: sometimes there is absolute intermission; but a very frequent, and, Mr. P. thinks, characteristic symptom is a want of accordance between the heart and the arteries in one or the other of the superior extremities, the heart beating violently, whilst the pulse in one arm may be scarcely perceptible. If the aneurism be previous to the giving off of the left subclavian, it may naturally affect the pulse at the wrist, but such aneurism can generally be recognised by auscultation. If the aneurism arises subsequently to the origin of the subclavian, when it is most difficult of discrimination, we do not see how it should occasion a difference between the pulse at the wrists.

One of the most curious features of thoracic aneurism is where it simulates acute laryngitis, in consequence (as supposed) of some pressure exercised by the tumour on the recurrent nerve. Mr. Porter relates an instance of this not *very* uncommon occurrence. As it is usually productive of embarrassment in practice, we shall introduce it.

"In July, 1837, I was requested by a professional friend to see a patient of his suffering from laryngitis, and, if I deemed it necessary, perform the operation of bronchotomy at once. It had been only of three days' standing: the symptoms were dreadfully urgent and pressing—so much so, that I thought I had never met a more formidable case; aud the idea of an aneurism never crossed my mind, as he had been attended by two physicians, one of them a most accomplished stethoscopist. I proceeded to operate without delay, and some notion may be formed of the urgency of the case when I state that I punctured the trachea with a trochar, rather than wait a few minutes for the purpose of controlling some hæmorrhage that was present. After the operation, which afforded immediate relief, the patient was removed to the Meath Hospital, where he died, suddenly, in three days afterwards.

The case had been one of aneurism of the superior part of the descending aorta, which terminated by bursting into the left pleura; and, as the difficulty of breathing had been relieved by the operation, I imagined it at the time to have been produced by spasm of the glottis, occasioned by pressure on the recurrent nerve. But I have since seen so many instances of spasm of this organ being produced by remote influences, and occurring under circumstances apparently so little in connexion with it, that I am unwilling to refer it to this, or indeed any particular cause, except remote sympathy—an expression which will cloak ignorance under an appearance of science as well as any other." 68.

With regard to *pain* as a symptom, Mr. Porter observes that he never knew a small aneurism not pressing upon any important tissue to be accompanied with any remarkable suffering, and instances are sufficiently familiar of patients having dropped dead in whom the existence of the disease had never been suspected, in consequence of their never having uttered a complaint. But it is very different when the tumour has attained a larger size, for then severe and lancinating pains are experienced, not only in the chest, but in parts very distant from it: thus patients complain of spasms, and stitches in the side—of acute soreness in the spine of the scapula—severe and constant headache, resembling hemicrania—and one of the most prominent symptoms is cramps in the muscles of the extremities, attended, occasionally, with great debility, amounting almost to a paralytic loss of power and motion.

As an instance of the devious course which thoracic aneurism occasionally takes; Mr. Porter mentions the case of a man who was admitted into the Meath Hospital, suffering from pains in the chest, and most of the rational symptoms of thoracic aneurism—these suddenly subsided, and were shortly followed by the appearance of a soft tumour in the left groin, not distinctly pulsatile, but evidently exhibiting a weak thrill. He died in three days, and on examination, an aneurism of the thoracic aorta was found—its sac had made its way downwards between the crura of the diaphragm, and had burst behind the peritoneum: the blood was then apparently conducted down along the psoas muscle and presented at the groin.

Amongst the signs of aneurism of the abdominal aorta, Mr. Porter, of course, discusses the "bruit de soufflet," one always valuable, if not absolutely characteristic. And he alludes to a hypothesis and suggestion of Dr. Corrigan's, ingenious if not satisfactory. Dr. Corrigan remarks:—"'if an aneurism be in a constant state of distention, equally as the arterial trunk with which it is connected, then there can be no gush of a diverging current of blood into it; there can be no vibration of its parietes, and there will of course, be no bruit de soufflet.' An aneurism of the abdominal aorta is peculiarly calculated for preventing the production of the sound in this manner, and the doctor purposes to remedy the defect by altering the patient's position. 'It occurred to me,' says he, 'that if I could relieve an aneurism of the abdominal aorta from this hydrostatic pressure, that keeps it constantly distended, thus preventing that gushing current into it which produces bruit de soufflet, this sign might become perceptible, and we should then be able, by its presence to diagnosticate aneurism of the aorta at a much earlier period than we have yet been able to achieve.' He places the patient then in a recumbent position, whilst employing the instrument. The obvious objection to this explanation is, that it supposes an aneurism to be in a constant state of distention, which it never can be as long as it contains fluid blood, and its sac is capable of yielding to the force of the heart's action: if it could be, the blood thus left in a state of repose would soon coagulate, and spontaneous recoveries be much more frequent and numerous than they are. The objection, however, is only theoretic, and with respect to the practical fact, I am quite aware that a bruit de soufflet may be heard in one position, whilst in another it may not."

*Treatment of Aneurism.*

Mr. Porter points out the natural modes in which aneurism may be cured—by inflammation and effusion of lymph within the artery—by gangrene—and by pressure of the tumour on the artery. Yet, says Mr. Porter, in the majority of cases, we seldom see one of them exemplified—the disease sometimes gradually subsides—sometimes suddenly disappears without our being able to explain the fact—and dissection has not hitherto satisfactorily developed the resources of nature in this particular. Mr. Porter is acquainted with a case in which an aneurism had proceeded so far as to have caused absorption of the sternum : the tumour pulsated externally with a violence that threatened its bursting almost momentarily; the patient lay on his bed, propped up with pillows, expecting nothing but the most miserable death; yet, without any obvious cause, and in despite of the prognostics of his attendants, the pulsation suddenly stopped, the tumour subsided, and he is not only alive but strong and active at this moment.

"A very interesting case occurred not long since at Stevens' Hospital: a man had an aneurismal tumour over the left clavicle, generally supposed to have been connected with the subclavian artery: various consultations were held upon the case, and operation was determined on when it was observed that the force of pulsation had somewhat diminished. I know not but that gentle compression might have been attempted, but the position of the tumour was so unfavourable that such treatment could have had no very decided influence on the disease; nevertheless, from day to day, the violence of the pulsation subsided—the size of the tumour diminished—and he left the hospital well.

I have seen one remarkable case of recovery which I scarcely know whether to designate as spontaneous or otherwise: it affords but little practical information, but is, nevertheless, too curious not to attract attention. In the month of December, 1831, I attempted to pass a ligature round the innominata for the cure of a very large subclavian aneurism, but failed, in consequence of the vessel being extensively diseased. The history of the case, and the steps of the operation, have been already detailed in the first number of the *Dublin Journal of Medical Science*, March, 1832, and therefore need not be repeated here: it is, however, interesting to state that the patient recovered perfectly. The aneurismal tumour disappeared entirely—the man's health and strength were so completely restored, that he was able to return to his former occupation, as a day-labourer in the country, and, I believe, he is alive and well at the moment in which I write. It is not reasonable to conjecture that the mere exposure of so large a vessel could have led to its obliteration." 77.

Mr. Porter insists upon a principle to be steadily kept in view in the surgical treatment of aneurism,—*that* it is a case of hæmorrhage, with the sole peculiarity that the blood which is withdrawn from the bleeding vessel still remains within the part or organ: the indication, therefore, to be fulfilled resolves itself into two parts—one, the arrest of hæmorrhage—the other, the removal of the effused blood. Now, it has been already seen that the first or immediate means by which hæmorrhage is controlled is by the application of pressure on the bleeding vessel—the second, or remote, the establishment of such a degree of healthy inflammation within it as will lead to its permanent obliteration. Such are the principles which guide us in all our treatment, both medical and operative. As a means of pressure we look to the coagulation of the blood contained in the sac. This sometimes cannot be attained, or, if attained, is insufficient. And by this fact hang some important features of prognosis.

"The aneurism which pulsates violently, and is increasing with great rapidity, is extremely unpromising, for in such there is a large aperture leading from the artery into the sac, and a large wave of blood, at every pulsation, disturbing that which had been previously effused, and preventing its coagulation.

Aneurisms which are very soft, and in which the contents of the sac are entirely, or nearly, fluid, are not likely to be benefitted by any treatment. There are some idiosyncrasies in which the blood scarcely seems to have any tendency to become coagulated at all, and I have seen large aneurisms, the sacs of which did not contain the smallest portion of a coagulum.

An aneurism, seated amongst soft and unresisting structures, is not one from which very favourable results may be anticipated: for even if the blood becomes coagulated, there may not be a sufficient force to press the clot against the bleeding vessel. This may obviously occur to aneurisms within the thorax, and I will have occasion to shew a very interesting example of this cause of failure in an aneurism placed more externally."

Mr. Porter proceeds to discuss the principles of treatment in internal aneurisms—rest and abstinence. Of course he describes the method of Valsalva, and adverts to the idea and practice of Dr. Stokes, the object of which was to diminish the quantity of the circulating blood, and, at the same time, increase that of the fibrine within it. He recommended small and repeated bleedings, but a diet the opposite to Valsalva's: it should be light and very nutritive, but, of course, free from any material of a stimulating nature. Mr. Porter admits the difficulty of the question and adds:—

"From observation on different cases of aneurism, I have been led to believe that there is a greater tendency to coagulation in the blood of one person than of another—in short, that there is a variety of disposition in this respect; but of the external marks, characters, or symptoms by which the presence or absence of such tendency can be recognised, I am wholly ignorant. At present I know of but one circumstance which certainly disposes the blood to coagulate—namely the abstraction of a great quantity of that fluid from the system, and, therefore, if I attempted a cure on this principle, I would employ large bleedings—so large that each of them should produce syncope or some other decided impression on the entire circulation: it is, however, but fair to state, that having made several trials of the plan, I cannot speak favorably of it as a means of permanent cure, although in other respects I may think highly of it. Unfortunately we do not often meet with internal aneurisms in their incipient or early stages, when we might more reasonably expect advantage from some decisive treatment—in such I have had no opportunity of making the experiment: but even in cases of long standing and large size, in which a cure might almost be regarded as impossible, I can speak of the efficacy of large bleedings, in affording temporary relief, with all the confidence derived from experience. In one remarkable instance, I had a patient with aortic aneurism bled to syncope several times, even to the extent of forty ounces of blood, and always with the obvious result of relieving the pain and difficulty of breathing, and enabling him to lie in any position, which, at the time of his admission, had been impracticable. At the moment in which

I write there is a patient in the Meath Hospital with an abdominal aneurism, of immense size and fearful strength of pulsation, that has been treated in the same manner with such marked relief from suffering that he has frequently solicited to have the operation repeated. An apprehension has been entertained by some that a patient thus suddenly reduced to a state of syncope might never rally, and actually die of, or be killed by, the operation; and, perhaps, such a casualty may have occurred, but nothing even approaching to it has ever happened within my observation." 83.

Mr. Porter touches on the experiments which have been made with the view of effecting coagulation of the blood in the sac by heat, electricity, galvanism, and the injection of certain fluids. He justly objects to these, that the blood is contained in the living body, and differently circumstanced from blood in a basin. "I have performed," says he, "numerous experiments of this description myself with the view of ascertaining the relative powers of different substances in producing a rapid and solid coagulation of fresh drawn blood, and never were experiments more completely devoid of any profitable result. Wherever the blood became rapidly *solidified*, the appearance, and (I suppose) the nature of the mass were changed, and a new substance formed in no way resembling a natural coagulum: the result seemed to be purely chemical, and subject to the laws that govern such operations in dead and inert substances. I have already stated that similar effects would probably not be produced on blood within the system, and even if they were, I feel convinced that the presence of this new material might prove highly irritating and injurious."

Mr. Porter observes that there are few circumstances that can justify our refusing to operate for an external aneurism. Yet when there is proof of the existence of organic disease in the heart, or of the presence of an internal aneurism, or if there are two or more external aneurisms indicating a general disposition to the production of the disease, the operation ought not to be performed; and yet, when the test of experience is applied to this precept, a vast deal of uncertainty will be discovered, occasioned partly by the difficulty of obtaining proofs of the existence of internal disease, and partly by the difference in the constitution of individuals. In a case of popliteal aneurism, Mr. Porter has known a patient to suffer from terrific palpitations, with occasional fainting fits, and other symptoms of disease of the heart, to the extent to render the propriety of an operation rather questionable; yet the man could not be left to die; the femoral artery was tied, and before the patient was removed from the table all these symptoms had subsided. Nor are two aneurisms a positive bar to operation. In the Meath Hospital (during the Winter of 1833-4,) was a patient who had a popliteal aneurism in each leg: he was operated on for both by Mr. M. Collis, an interval of six weeks being allowed between the recovery after one and the performance of the second, and the result was in every way satisfactory. The man however was lost sight of.

Mr. Porter considers the question of amputation one that must occasionally be raised. If the bone is extensively carious, this is requisite. And it may be so, if the aneurism has grown to a large size, when gangrene might follow the ligature of the artery. This has usually, says Mr. Porter, been attributed to the obstruction of the main vessel, and the inability of the collaterals to convey a sufficient supply of blood; but there are no grounds or reasons to countenance such a supposition—first, because the contrary has been proved by experience, in thousands of instances; and, secondly, because gangrene, when it does occur, is not of the dry and shrivelled kind that seems to arise from diminished circulation and imperfect nutriment, but humid and moist, like that occasioned by a congested state of the veins, and an accumulation of blood within them. If, then, he adds, the tumour is very large, and has already interfered with the return of the venous blood—if the limb has become œdematous and painful —if the superficial veins are enlarged, and ramify numerously under the surface of the skin—or, if the limb is deficient in animal heat—there will be reason to dread the results of tying the artery; and, probably, many cases of mortification that have either not been accounted for, or attributed erroneously to other causes, might, on closer investigation, have been traced to this. It is also worthy of observation, that in cases of large popliteal aneurisms, although a cure of the chief and important disease may have been effected, the patients are liable to abscesses of the limb, and to tedious and inveterate ulcers of the leg for a long time afterwards, or it may be, during their lives.

When the sac contains too great a quantity of blood for the absorbents to remove, this becomes a source of inflammation and of suppuration. This may happen about six weeks after the occurrence of the ligature. Amputation, however, is not absolutely requisite on this account, for, except in some singularly unfortunate cases, the artery has been obliterated before the sac becomes inflamed. There is no danger from hæmorrhage on opening the tumour; and the proper mode of treating such a case would be, to make a free and extensive incision into it, discharge all the matter, turn out every particle of coagulum, and then apply pressure, in order to promote the agglutination of its opposite sides. The sacs of two subclavian and one carotid aneurism under Mr. Porter's care suppurated, were thus treated, and recovered without trouble, and even without an unpleasant symptom.

Mr. Porter does not think that the earthy or bony deposition in the arteries of aged persons constitute any valid obstacle to operation. In the healthiest artery the internal and middle coats are divided, and the ligature is sustained by the cellular one alone, and no more can happen with the artery of the aged person, because this earthy material has no connexion with the cellular tissue. It might indeed happen that a vessel thus circumstanced would not go through the process of adhesion and obliteration, and therefore there might be risk of secondary hæmorrhage; but there can be none of its immediate occurrence. Mr. Porter has operated on an artery of this kind without any unpleasant result, either immediate or remote, and would have no hesitation in doing so again, if urged by the necessity of the case.

Mr. Porter proceeds to examine the modes of treating an external aneurism, viz. pressure—ligature on the cardiac side of the tumour—and ligature on the distal side of it.

1. Mr. Porter mentions some remarkable cases of the good effects of compression.

"Some years since, a man suffering from aneurism, was admitted into the Meath Hospital. The tumour was situated low down in the popliteal space, and was large, being fully the size of a turkey's egg. The limb was semiflexed, and could not be extended: pain very considerable, together with a sensation of numbness and tingling in the foot: tumour not compressible, at least pressure influenced its size but slightly: it was hard, and did not diminish in bulk when the femoral artery was compressed, which, however, stopped the pulsation. With a view to humour the patient, until he could be persuaded to submit to an operation which I conceived to be absolutely necessary, I rolled a bandage round the entire limb, from the toes upwards. This, as the idea of treating the disease by compression had never been contemplated, was very loose; nor had I the least notion that the tumour could have been influenced by it, one way or the other. But, on my visit the next day, the aneurism was gone. Within an hour after the application of the bandage, the patient experienced some pain in the tumour, which soon became excruciating, and continued the entire night. In the morning the tumour no longer pulsated—it had become solid and firm, and eventually the disease was cured. On another occasion a man was admitted into the same hospital, under the care of Mr. M. Collis, with a popliteal aneurism, the history of which I do not recollect with very great accuracy, except that it was rather of a large size, not compressible, and there seemed not to be much fluid blood within the sac. A bandage was applied to this in a similar manner to that in the former case: it caused immense pain; and in the following morning the pulsation in the tumour was no longer to be remarked. It, however, reappeared after a little time, but so very indistinctly that it was a questionable matter whether the sensation was not communicated from the finger of the examiner, and not from the tumour. The application of the bandage was persevered in, and, in the course of a very few days, no doubt could be entertained of the cure.

Such instances of inexplicable recovery are extremely rare, and, as examples of singular good fortune, are rather to be hoped for than expected; neither can any principle of practice be established on them; but I have occasionally seen compression used in another way with such great success as would, with most practitioners, sanction its adoption in every similar case. The cases I principally allude to were treated in Stevens' Hospital by my friend, Mr. Cusack. As some of them have been published, it will be unnecessary here to state more than that the disease had been the consequence of wound—that the patients were young and healthy men—that the compressing force was evenly applied over the surface, and gradually increased, having been very gentle at first—and that the treatment was assisted by the topical application of cold, and the internal administration of digitalis. Within a very short time past, a patient in the same hospital, and under the care of the same distinguished surgeon, was treated in a similar manner, with the most signal success, for an aneurism of the anterior tibial artery, caused by the wound of a chisel." 100.

But compression has not practically answered on the whole, for the artery is often not favourably situated for it, and the sac might be ruptured by it, whilst the pain of efficient compression is not unfrequently unbearable. Mr. Porter remarks that it may certainly be warrantable to try it where the disease has been the consequence of a wound, and the vessel may be presumed to be otherwise healthy; but even then the size of the tumour becomes the subject of important consideration. If an aneurism is small, the compress may possibly be so placed as to lie directly on the cicatrix of the sac, thereby supporting it, and preventing its contents from being diffused; but if it is large the situation of the puncture will probably be towards one side, and then pressure on the centre will rather have a tendency to force it open and drive out its contained blood amongst the cellular tissue of the limb. It is difficult to lay down any rule, and each surgeon will probably determine for himself. Mr. Porter expresses his own opinion :—

"It has been my lot to witness some cases of circumscribed aneurism rendered diffused by pressure; and having seen the patient's distress and danger, the painful, the tedious and difficult operations he had to undergo, and the hazard to which life was exposed afterwards; and having well weighed the different contingencies that may occur, I have come to the conclusion, in my own practice, not to resort to compression, unless there was some insurmountable objection or obstacle to the employment of the ligature." 102.

The *Treatment of Aneurism by Ligature* next occupies Mr. Porter's attention. He imagines that the process by which aneurism is cured by the application of a ligature at its cardiac side, is not the same in the false aneurism and in the true: at least it is difficult to explain the varieties met with, or any other supposition. In the false, the great object to be accomplished is the removal of the impulse of the heart from the blood contained within the sac for a sufficient time to allow of this reservoir becoming slowly and gradually filled with blood, and for that blood to become firm and coagulated. This coagulum, then, is pressed upon or against the wounded and bleeding vessel, and produces those consequences within it that terminate in its obliteration; the blood, if the case proceeds favourably, is afterwards absorbed, and the sac, in process of time, is converted into a solid piece of ligamentous substance similar to that into which the arterial trunk has degenerated. The completion of this process requires different periods of time, according to the size of the vessel, the dimensions of the sac, and the quantity of fluid blood it can contain, but is always accomplished long before the separation of the ligature.

Speaking of the subsequent removal by absorption of the coagulum within the sac, and of the occasional occurrence of suppuration in it, he observes that, in several instances, he has evidently traced this unpleasant accident to a too frequent and injudicious examination of the tumour—in fact, to handling and compressing it to see if it was rapidly subsiding. It is painful, and may appear ungracious even to seem to repress the laudable zeal for inquiry and investigation that ought to be the characteristic of every student as well as of every surgeon; but in cases of aneurism, an over-weening curiosity may be worse than useless—it may be injurious, or even destructive to the patient.

Mr. Porter does not think that the same principle of cure obtains in the true aneurism and in the false. In most instances of aneurism, particularly when the tumour has attained any considerable size, the wall of the sac is so thickened, and all the structures are so matted together and confused by depositions of lymph and fibrine, that the appearances are rendered most deceptive, and a very patient investigation will be necessary to develop the morbid changes. But there is one circumstance which, when observed, at once demonstrates that the aneurism has been produced by dilation, and not by rupture or ulceration. It is when the wall of the sac contains depositions which are only met with in the structures of the artery, such as the peculiar earthy scales that are formed in the vessels of aged persons, and the soft steatomatous material that may be the product of unhealthy inflammation at any period of life. In opposition to the opinion of Scarpa, Mr. Porter entertains no doubt that there is a pathological condition of an artery producing all the symptoms of circumscribed false aneurism, and apparently curable by the same means—that is, by the application of a ligature at its cardiac side. It seems to be formed by a dilation of the fibrous and cellular coats, and the absorption of the internal lining; and thus is so far a true aneurism as that the artery is uniformly dilated around its entire circumference, and so far a false one, that the internal lining membrane has been removed. Mr. Porter gives a case in illustration. In March, 1835, a man was admitted into the Meath Hospital, with popliteal aneurism in each ham, one of which had existed for several weeks; the other was of recent occurrence. The limb in which the larger and older one was situated was first made the subject of operation—the femoral artery was tied, but the patient died on the sixteenth day afterwards, the ligature on the vessel still remaining firm and undetached. Dissection shewed that both these aneurismal sacs had been formed by the entire and equable dilation of the fibrous and external coats of the arteries, and that the lining membrane had in both instances been absorbed. Mr. Porter relates another case, for the purpose of exhibiting the principle on which this form of aneurism is probably cured by operation. A man was operated on by Mr. Collis, in the Meath Hospital, for popliteal aneurism, on the 22d of January, 1831. The ligature came away on the seventeenth day—the tumour diminished; in short, everything went on well, and the patient left the hospital perfectly cured. So far as the aneurism was concerned, he remained healthy and free from inconvenience until his death, which happened in March, 1835, from fever, and such an opportunity for pathological inquiry was not neglected. The tumour, which had been originally of the size of a turkey's egg, was found to have diminished to little more than that of a walnut; externally it felt hard, and as if solidified. On being cut into, however, neither artery nor sac was obliterated—the latter being occupied by a coagulum of a deep red colour, through the centre of which was a canal of a sufficient size to allow the blood from the portion of the artery above the tumour to flow freely into that below it. It seemed as if the current of blood through the sac had never been interrupted, the only effect of the ligature having been the removal of the impulse of the heart from it.

Mr. Porter observes, that whenever an artery is dilated, either wholly or in part, the dilated portion is uiformly found after death filled with coagulated blood: it seems that under such circumstances the fibrous coat is so far weakened as to be incapable of emptying the vessel after it has been filled by the last stroke of the heart, and the blood that is within it remains there, and Mr. P. imagines that something of the same kind takes place during life, when the impulse of the heart has been removed by the interposition of a ligature. He details a case of aneurism of the femoral artery, and proceeds :—

"From this case I infer—1st, that the contractile force by which an artery is emptied immediately previous to death, exists in the vessel, and is wholly independent of the heart, inasmuch as any assistance to be derived from the latter had been here intercepted by the ligature; and, 2dly, that that force (whatever it may be) did not exist in the dilated portion of the vessel. Now, if reference is made to the manner in which blood circulates through a dilated vessel during life—that the dilated portion is always full, and is expanded during the diastole, by the influx of a very small wave of blood sent by the heart, and is diminished by the exit of a corresponding portion, caused more by the elastic resistance of the parts covering the artery than of that of the vessel itself—it will not be difficult to perceive that the circulation of the blood through an artery, the vital qualities of which are thus impaired, the constant disturbance of the fluid within it, and the prevention of its coagulation, depend upon the heart, and that the blood will stagnate and become coagulated when the impulse derivable from that organ is lost. It is thus that I conceive true aneurism is cured." 122.

We must confess that we are not altogether satisfied of the correctness of Mr. Porter's notions with regard to true aneurism. And we suspect that his views will not be received without demur.

Mr. Porter touches on the ligature of the artery on the distal side of the aneurism. He thus sums up his own convictions. "As to the ligatures of the subclavian and carotid, for the cure of an aneurism of the innominata, I never allowed my mind to dwell on it for a moment, believing that it was necessary that both branches should be tied; and entertaining the opinion I do, that the ligature of the subclavian, in its first stage, before it reaches the scalenus, cannot, for anatomical reasons, ever be practised with success. It appears, however, that in such a case the ligature of the carotid alone has answered the purpose, and so far experience seems to be in direct opposition to theory. Notwithstanding all these authorities, I confess I still find it difficult, in my own mind, to place confidence in this operation, or to recommend it, except in cases where, from the size and situation of the tumour, it must be impracticable to apply the ligature at its cardiac side; then, indeed, the patient would be entitled to whatever chance it might offer, however meagre that chance would be." We fancy that the majority of surgeons will acquiesce in this opinion.

*Diffused Aneurism.*—Mr. Porter draws a distinction between "diffused aneurism" and "traumatic aneurism." The latter term he limits to the case, where there is an external wound so leading down to, and communicating with, the injured artery as to permit the escape of a portion of the blood from it. This latter is the essential condition of traumatic aneurism. Thus, the affection may have been produced by a wound, yet if that wound is healed, or not being healed, if it is very oblique, or if there is any circumstance that prevents the blood from passing out of the limb or part, it falls not under the appellation of traumatic aneurism. In either case, says Mr. Porter, there is always very great danger, partly arising from the loss of blood, but more particularly from the presence of the blood within the limb inducing a state of mortification; but this latter peril, being always proportioned to the extent to which the diffusion has reached, may be considerably modified by circumstances. Thus, if the wounded artery is firmly bound down by a fascia, or if there is any other obstacle to the free and rapid passage of the blood upwards and downwards through the limb, the growth and progress of the disease may be extremely slow, and it is only when the fascia has sloughed, or the resistance is otherwise removed, that the diffusion becomes extensive, and the patient's danger imminent. And the worst feature of the case is, that the nature of the disease may not, and probably will not, be recognised until it is too late. Mr. Porter mentions an interesting case.

A man received a blow on his leg, by the fall of a crate of glass on shipboard, whilst on his passage from Liverpool to Dublin; he came to the hospital complaining of deep and dreadful pain in the limb; but, on examination there was nothing abnormal to be found, no swelling, discoloration, tenderness, or lameness, and consequently very little attention was given to the case. In this state he went about during a period of six weeks, from one institution to another, at some regarded as an hypochondriac, at others, perhaps, as an impostor, but at all completely overlooked, when, at the end of that time, he experienced a sensation as if something had given way within the leg, which immediately began to swell, and ran so rapidly into gangrene that amputation was performed on the following day. He died, and, on dissection of the limb, after removal, the posterior tibial artery was found ruptured, and a large quantity of blood under the deep fascia that lies behind it. A ragged sloughy aperture was seen in this fascia, and the limb in every direction extensively injected with blood. It seemed as if the artery had been injured by the original blow, and had bled under the fascia which subsequently gave way and burst, and then mortification rapidly ensued.

The treatment of diffused aneurism naturally occupies him. Mr. P. thinks that there are three varieties of case connected with diffused aneurism that may require each a distinct and separate mode of treatment.

1. Mr. Porter considers the wounded and bleeding artery as a point of secondary importance in coming to a decision as to the line of practice to be adopted, the chief being the quantity of blood effused; for if it is very great, even although mortification may not have actually taken place, yet it is quite possible the limb may be in such a condition as to render it inevitable: or supposing otherwise, and that there is so little apparent ground for this apprehension that the artery only is secured, still, if there is much blood, the probability is, that it will act as a foreign body, or perhaps that it may putrefy—that large abscesses and profuse suppuration will result, involving the necessity of deep and extensive incisions in a limb otherwise severely injured, and that a rapidly destructive hectic would be established. To determine the propriety, or otherwise, of amputation, all the circumstances must be taken into the account, and what should cause a leaning against amputation is the fact, that when performed for aneurism, it has been singularly unsuccessful.

2. "The next case for consideration is that in which an attempt is to be made to save the limb, and here the question for decision is whether the vessel may be tied at a distance, as in circumscribed aneurism, or whether it must be cut on and secured above and below the seat of injury: and here the quantity of blood effused must again be taken into account, as well as the presence or absence of an external wound. If there is no such wound, and the quantity of blood is not so very great as to dissipate every hope of its being absorbed, I see no reason why the Hunterian operation should not prove successful. The principle on which a cure is accomplished in diffused aneurism without a wound ought to be precisely the same as in the circumscribed, namely, the wound in the artery must be subjected to a degree of pressure sufficient to effect the obliteration of the vessel at that spot, and this should be perfected by the coagulum in the one instance as well as in the other." 137.

Of course it is desirable to avoid, if possible, cutting into a limb so gorged with blood, or *on* an artery so circumstanced. And in the tenth number of the *Repertoire d'Anatomie et de Physiologie Pathologiques*, there is a memoir by Baron Dupuytren, in which two cases are detailed, one that was under his own superintendence, in the Hotel Dieu, and the other under that of Delpech, at Montpelier, which go far to prove that the laceration of an artery by a spiculum of bone may be cured by the Hunterian operation.

3. But there is still another case, and let us suppose a limb into which a quantity of blood has been extravasated, not sufficient to cause a gangrene of the part, but still too abundant to admit a hope of its being absorbed. Here, I apprehend, the surgeon has no choice—he must cut down, turn out all the coagula he can reach, in this way getting rid of that which would be a subsequent source of irritation, and then tie the vessel above and below the aperture.

Mr. Porter touches on another point. If the diffusion has been caused by the rupture of a circumscribed sac of an idiopathic aneurism, it will be the more objectionable to cut down upon the injured spot, because there is so far presumptive evidence of the artery not being healthy in that situation, and consequently the risk of secondary hæmorrhage will be the greater.

4. For "traumatic aneurism" Mr. Porter advises the cutting down directly on the injured vessel, and tying it above and below the wound. Mr. Porter illustrates his position by a reference to the case of the brachial artery wounded in bleeding. He draws a practical distinction between the case where the artery is wounded without much effusion of blood into the parts around, and that where the effusion is considerable. Of these cases, one is only hæmorrhage, the other diffused aneurism. In the former there can be no reason why compression should not be tried—the artery is healthy, its position preserved, and its condition, in every respect, favourable; and, if properly and firmly bandaged, the wound may unite well, and the hæmorrhage be perfectly controlled.

If pressure, *judiciously* applied, fails, there is still a chance that the aneurism may prove circumscribed, and admit of subsequent treatment by the Hunterian operation. But in the actual traumatic aneurism pressure is not likely to succeed. Suppose it to be attempted, where is it to be applied? The artery in all probability has been pushed by the effused blood out of its position, and we know not where it is placed. And how is it to be maintained? To be of any use it must be even, firm, and unyielding, and the pain it would occasion must be absolutely intolerable—most patients would remove the bandage in despite of any warning to the contrary. Such are the objections to pressure even at an early period; but when the aneurism has become decidedly diffused, it is of course out of the question.

"It may possibly be objected, that in many cases of traumatic aneurism, a milder mode of treatment might be adopted, for that success has attended the application of a ligature on a distant part of the artery. I concede the point. If the obliquity of the wound, or its smallness of size, or any other circumstance connected with it, offers such obstacle to the flow of blood, that on the removal of the impulse of the heart from that part of the circulation, the external bleeding ceases, then the aneurism, immediately on the application of the ligature, loses its traumatic character, and is no longer subject to its laws: but how do I know, previous to an operation, that such is the exact nature of the case with which I have to deal? I see before me a wound, 'permitting coagulated blood to protrude, and fluid blood to trickle,' can I tell that these phenomena are produced and maintained by the impulse of the heart alone, and will cease when that influence is removed? No! such knowledge is not mine, and if in the spirit of empiricism, I practice the Hunterian operation and succeed, I have just the consolation of reflecting, that I acted rightly on wrong principles, and cured my patient, by exposing him to a double and therefore an unwarrantable danger. Again, if after the ligature has been tied, a sufficient degree of pressure is applied to lay the sides of the bleeding artery together, occasion its inflammation and subsequent obliteration—in short, if external artificial pressure can be made to effect all that which the resistance of the skin, and fascia, and other superincumbent structures would have accomplished in a less injured limb—then I conceive it possible that the removal of the impulse of the heart may be followed by a cure. But how do I know that I can apply such pressure, or, being applied, that it can be borne?" 144.

*Causes of Failure of the Operation for Aneurysm.*—Mr. Porter first touches on the return of pulsation in the sac. In proportion to the facility with which the blood enters this, is the cure of the disease put in jeopardy. The phenomenon in question may arise from different causes, some of which are known—others, perhaps, remain to be discovered. One is the existence, by an irregular distribution, of two trunks in the limb, both conveying blood to the aneurismal tumour. Sir C. Bell had a case of popliteal aneurism in the Middlesex Hospital, in which, just below the origin of the profunda, the femoral artery divided into two branches of nearly equal size, which ran parallel to each other, until they arrived at the spot where the artery perforates the tendon of the triceps muscle, and there they united again. Only one of these was tied, and although the pulsation ceased for a moment, yet it soon returned, and never disappeared until the patient's death, which happened a few days afterwards from erysipelas. A preparation of a similar distribution is preserved in the museum of the Royal College of Surgeons in Dublin, and Dr. Todd states that another is to be seen in St. Bartholomew's Hospital, London.

Another case, in which pulsation will be likely to re-appear in the tumour, is where one or more large branches arise from, or otherwise communicate with, the sac. The possibility of this occurrence has been abundantly proved in internal aneurism, where it shews that the morbid condition of the vessel is dilation and not rupture or ulceration, but Mr. Porter is not aware that dissection has shown it in external aneurism.

Mr. Porter conjectures, that tying the ligature too loosely may be another cause. A few years since, the femoral artery was tied by Mr. Cusack for popliteal aneurism. The patient was excessively restless and turbulent, and it was with the greatest difficulty the operation could be completed at all. In the course of a few hours pulsation re-appeared in the tumour, and remained, for a few days, gradually dying away and becoming feebler, but the case nevertheless progressed favourably, and at little more than the usual period the ligature came away. When examined, it was evident the noose had been tied loosely; it could easily allow a tolerably large goose-quill to pass through it. About the same time Mr. P. saw a similar case, except that in this latter the noose was left large designedly, with a view that the impulse of the heart only should be removed, without absolutely preventing the passage of the blood. In this instance the appearances were very curious. When the patient's limb was bent, and the artery, as it were, relaxed, pulsation was not only evident, but tolerably strong in the tumour; when, on the contrary, the limb was extended, the pulsation disappeared. The noose of this ligature also separated slowly, and when removed was found to be very large.

The next circumstance is the existence of such an extensive and free communication, by anastomoses, as will convey to the tumour, by a circuitous route, the impulse which the ligature was intended to remove. It is probable that this condition obtains, with respect to aneurisms of the internal carotid in the neck, and that the free and extensive anastomoses through the vessels of the brain in their natural and normal state, will be quite sufficient, to interfere more or less completely with the cure. Mr. Porter relates three cases in point.

There is an anatomical peculiarity in the internal carotid, unfavourable to the exercise of pressure on the artery by the coagulum in the sac. Internally, or towards the pharynx, it has nothing but the mucous membrane, the constrictor of the pharynx, some very loose cellular tissue, and the twigs of the superior laryngeal nerve; thus the aneurismal sac has ample room to grow and increase inwardly, and, consequently, the pressure it is forced to make on the opening in the vessel may be so trifling as not in any way to lead to its obliteration. Mr. Porter points out the necessity for examination of a carotid aneurism by the mouth.

A young girl, named E—— M———, was admitted into the Meath Hospital, with a varicose aneurism situated at the angle of the jaw, and extending downwards a short way along the course of the external jugular vein. It became very large and prominent when pressure was made on this vessel below, so as to interrupt the current of blood; it became then excessively painful, and exhibited the usual thrilling sensations, both to the finger and the ear; but when looked at from the mouth, a strong and continued pulsation, together with considerable tumefaction, was visible to every eye.

A probable reason of the non-success of the operation in some instances, is constitutional idiosyncrasy preventing the coagulation of the blood. The following appears to be a case in point. Some years ago, a man was admitted into the Meath Hospital, having received a stab of a sharp-pointed shoemaker's knife, about an inch below the right sternoclavicular articulation, by which the internal mammary artery and vein were wounded. These vessels poured out their blood continually, and in such abundance, into the cavity of the pleura, that the lung became dreadfully oppressed, and it was deemed advisable to perform the operation of paracentesis, on the fourth or fifth day after the receipt of the injury. The wound made in the operation was large, in the expectation that it might facilitate the escape of any coagulum, but the precaution was found to have been unnecessary, as the blood had remained in a perfectly fluid state, and flowed away with great facility. The quantity of blood thus lost was enormous, it must have amounted to some quarts, and as the wounded vessels still continued to bleed, it is scarcely necessary to add, that the patient soon died. On examination after death, a good deal of fluid blood was found in the cavity of the pleura, but not a particle in a state of coagulation. But, in fact, there are many cases on record more striking than this.

"There still remains one condition more to be noticed, in which pulsation has been observed to return in the tumour after a ligature has been applied on the arterial trunk, namely, where it appears in consequence of, or, at least, in connexion with, inflammation of the leading vein. It is perhaps, not easy to place these occurrences, in the relation of cause and effect, in the clear light of demonstration; neither is it of much importance, in the study of aneurism, for, when this calamitous accident takes place, the condition of the sac will form but a secondary consideration; still, the observation is new, and may be interesting in a physiological point of view. The peculiarity of the phenomenon in this case is, that it occurs after the inflammation has been established, that is, at a much later period than when produced by any of the preceding causes; that it increases in intensity along with it; and that examination after death exhibits no explanation of it except the one advanced. I apprehend the cause of it to be shortly this, that blood does not circulate through the inflamed vein—that congestion, consequently, occurs in the minor branches and capillaries—and that, as a result of the resistance thus offered to the circulation, a reflex action is produced in the arteries, just as in a case of congestion from inflammation or any other cause." 173.

*Case.*—On the 4th July, 1840, in the operation of securing the femoral artery for the cure of a popliteal aneurism, the vein was unfortunately pricked, and a small portion of it included in the ligature along with the artery. The case progressed pretty nearly as all such cases do, as unfavourably as possible, and the patient died, on the fifteenth day after the operation, of inflammation of the vein and its consequences. Pulsation re-appeared in the sac on the 10th day, and gradually increased in violence until the man began to sink a few hours before his death.

On dissection, the vein was found extensively inflamed, but the inflammation had passed more freely down the limb, where it extended even beyond the popliteal space, than upwards, for it was limited in that direction at the groin. The vessel appeared enlarged—its coats thickened and solidified—on being slit open, the lining membrane seemed to be in a state of slough—it was of a muddy brown colour and bathed in a quantity of foul and fetid sanies. Masses of soft broken lymph were seen filling up the entire of the canal in several places, but there was no clot of blood, nor any discoloration of the lymph, to indicate that it had been in contact with blood since the time of its formation. Placed in connexion with the œdema of the limb, the enlargement of the superficial veins and other symptoms that occurred during life to indicate an obstructed circulation, it was the opinion of those who examined the limb, that blood had not circulated in the vein from the time it became inflamed.

*Secondary Hæmorrhage* is the next subject discussed by Mr. Porter. He dwells upon its difficulty, and on the uncertainty which envelops the causes of this formidable accident.

Practically, says Mr. Porter, we meet with *secondary hæmorrhage* under two conditions—one, where there has been previous bleeding, which, being controlled for some time, bursts forth again: thus, if a bleeding vessel that had been tied, or a surface to which the cautery had been applied, should bleed, or even if a hæmorrhage that had seemed to have been stopped by the sanative powers of Nature alone, should appear again, this bleeding is termed secondary. The other, where there has *not* been previous bleeding, but where a ligature or some other form of compression has been applied to an artery, in order to the cure of an aneurism situated in some distant part. " Now," adds Mr. Porter, " in the first of these, all the cases of bleeding that occur at a very early period should be termed 'primary.' If a ligature has been loosely and imperfectly applied, and slips from its hold on the vessel—if a patient has been put to bed after an operation without all the wounded arteries being secured—if compression has been resorted to, and the bandage or other contrivance has yielded—or if the cautery has been applied sparingly and insufficiently, there is as little reason for considering the hæmorrhage as secondary, as there is for compassionating the surgeon, to whose *insouciance* the accident is attributable."

It appears to us that were Mr. Porter's suggestion acted on, there would be inconvenience and difficulty. The smaller vessels of a stump which are usually sealed by Nature at once, bleed in twelve or twenty-four hours after the performance of an operation. Surely this is as much secondary hæmorrhage, as if, on the separation of a ligature, the natural process has not closed the vessel. It is right, of course, to be acquainted with all the various circumstances that give a disposition to such hæmorrhage, and to discriminate that which occurs early, from that which happens at a later period. And this we think the best-informed surgeons do.

It is not the least important feature of hæmorrhage, whether primary or secondary, that it evinces great capriciousness in its cessation. Ruspini's styptic, and probably other more orthodox drugs, owe no trifling part of their reputation to this. The bleeding stops, perhaps all at once, and the nostrum gets the credit of it.

*Case.*—A boy, attempting some improper liberties with a female servant, was struck by her with a knife in the superior part of the thigh, and the profunda artery wounded: the bleeding was immediately controlled, but, after nine days, burst out again, welling forth without impetus, and coming from the inferior section of the artery. Pressure was applied, but inefficiently, for in a little time he bled again, and, after several recurrences of hæmorrhage, he was reduced to so deplorable a condition that something more effectual should be done. The application of a ligature on the bleeding vessel was out of the question, for, lying deep on the bottom of a granulating wound, its mouth could not be discovered: he was placed on the table, in order to have the iliac artery tied, when some person remarked that he had not bled since the previous evening—he was replaced in bed and never bled afterwards.

Mr. Porter cannot find a satisfactory instance of very early secondary hæmorrhage from the application of a single ligature, as in a case of aneurism.

The second form of consecutive hæmorrhage may be again subdivided into two classes essentially differing from each other in their origin, progress, and terminations, one occurring at a period near to, but somewhat earlier than, the time the ligature ought naturally and healthily to come away—the other, at a much later period, when the cord has been long separated, and, perhaps, a reasonable degree of confidence might have been entertained that the patient had surmounted every danger. The first of these is by far the most frequent of occurrence, and is sometimes, but not always, preceded by febrile symptoms and other indications of the coming event. The artery has usually been completely divided by the cord which may have come away. Mr. Porter has seen the blood flowing from the inferior segment, whilst the ligature was still firm and fixed upon the upper. It is always the inferior portion that bleeds. Though few patients die of the actual hæmorrhage, yet, either the extensive and successive bleedings, or the means it may be necessary to have recourse to in order to control them, or the wretched state of constitution that predisposed to the disease, and is daily aggravated by its continuance, each or all of these may lay the foundation for future ills. There have been several such *fortunate* cases in the Meath Hospital. In some mortification took place, probably induced by pressure on the vein, and one of these was only saved by amputation performed while the gangrene was still spreading. Others, again, at a more remote period, suffer from abscess, ulceration, periostitis, caries, mortification, and a host of other maladies involving the loss either of limb or of life. Mr. Porter has never seen a case of secondary hæmorrhage from the femoral artery that there was not erysipelatous inflammation and abscess on the outside of the thigh near the great trochanter. Unquestionably, it is not always easy to connect these accidents with the consecutive hæmorrhage by accurate pathological reasoning; but they occur too constantly to admit any doubt of their relation.

Mr. Porter explains the occurrence of secondary hæmorrhage by the existence of unhealthy, in lieu of the proper healthy inflammation of the artery. As a consequence lymph has not been thrown out. He adds,—

"The most curious part of the question is, why these two forms of inflammation should occur in the same individual; yet, experience proves that secondary hæmorrhage only takes place from the segment below the ligature, and I have verified by dissection the fact that a different kind of inflammation takes place at the cardiac and the distal side of the cord. A man died on the sixteenth day after the operation for popliteal aneurism, and whilst the ligature still remained undetached from the artery. The vessel was carefully removed from the body, and on being slit up, the lining membrane of the portion at the cardiac side of the ligature was of a pale yellow colour, and nearly of its natural appearance, with the exception of one or two broad spots of a very light pink colour. A large coagulum extended upwards from the seat of the ligature, the base of which was attac[illegible]d to the lymph situated there. The ligature was still firm, but on attempting to tear it away, the lower portion of the vessel easily separated from it, leaving it still firmly fixed on the upper section—a circumstance that explained a fact I had frequently witnessed, that of secondary hæmorrhage occurring before the final separation of the cord. *Below* the spot where it had been tied, the vessel appeared to be of a deep pink colour, approaching to carmine, the seat of which colouring matter was in the cellular tissue, between the fibrous and internal coats. This cellular substance seemed to be hypertrophied and largely congested with blood, while it caused the lining membrane to be thrown into transverse rugæ, or folds. On pulling off the membrane, it was pale, transparent, and colourless—devoid of proper vascularity; and, on looking along the slit side of the vessel, the fibrous coat and the internal membrane were seen like white lines, with the congested cellular tissue between them. There was not a particle of coagulum, either of blood or lymph, in any portion of the vessel below the ligature." 184.

The following opinions will not be deemed quite orthodox. Mr. Porter speaks of the denudation of the vessel in the operation, a reputed cause of hæmorrhage.

"It has been already observed," says he, "that some of the most clumsily performed operations have had the best success, and *vicê versâ*—a circumstance that could not happen if the denudation of the vessel was of such extraordinary consequence. Again, in considering the subject pathologically, we find that ulceration is not a diseased process to which the arterial structure is prone, except when it occurs internally, and as a sequela to the inflammation and steatomatous deposit already so extensively noticed. An artery will lie perfectly denuded and white at the bottom of a phagedenic ulcer in the groin for days, before it gives way and bleeds; and when it does, it is not by ulceration, but by the formation of a slough, on the separation of which the blood bursts forth rapidly, and in a manner totally different from the phenomena of secondary hæmorrhage. Lastly, if this denudation was to have any injurious effect, it should be by cutting off the vascular supply from which the vessel derived its nutriment, and the consequence of that ought to be sloughing, and not ulceration; and, in either case, both sections of the artery should suffer alike, whilst it is known that the bleeding always comes from the inferior. Moreover, although experiments on animals cannot have any decisive importance attached to them, yet they may be noticed when they bear remarkably on any particular point. Haller and John Hunter, Sir E. Home, and, in this country, Sir P. Crampton, dissected off the external and fibrous coats of arteries with a view to ascertain whether aneurism could be produced by a protrusion or hernia of the internal one. In such an experiment, this membrane must have been deprived of all connexion with adjacent parts, and cut off from all vascular supplies. Why did it not die and slough away?—at all events, why did it not ulcerate, if this pathological explanation of secondary hæmorrhage be the true and correct one? The answer is simple and plain—because, in order to this result, a particular kind of inflammation must be established in the vessel, of which the artery of an animal is not susceptible, and which, although it may occur in man, is certainly not produced by the denudation so much spoken of and relied on. In these pathological observations let me not be misunderstood as advocating careless or clumsy operations; for, if operating myself, I should not wish to expose more of the vessel than would barely permit of the passage of the ligature around it; but I cannot, at the same time, shut my eyes to the truth, and admit as the solution of a phenomenon a fact so obviously insufficient." 186.

We are not quite sure that the preceding reasoning is conclusive. In the first place, it is certain that to denude the artery extensively *must* interfere with its vascular supply—in the second place, such interference cannot but tend to impair the vitality of the vessel—in the third place, there is no reason why ulceration should not ensue, as well as sloughing—in the fourth place, the matter of fact would seem to be that secondary hæmorrhage *was* more frequent than it *is*, and vessels *were* more bared in operations than they *are*—in the fifth place, experiments on animals are peculiarly unsatisfactory in such a question—and in the sixth place, the circumstance of some patients escaping in spite of denudation of the artery, proves rather the strength of their constitutions than the safety of any such practice.

Mr. Porter alludes to the plan of Mr. Abernethy, that, namely, of applying two ligatures to the artery, and dividing it between them. He says,—"What could be done more entirely calculated to avoid these influences than this operation? and yet, when we test it by the results of experience, we find it valueless; for it cannot be questioned, and the observation is supported by the authority of Dupuytren, that as many cases of consecutive hæmorrhage follow this mode of operating as any other; if permitted to speak from my own experience, I would say—more. Here, then, both the ligatures are placed as remotely as possible from each other—no part of the vessel is denuded or disturbed, except the intercept between them—and, when this is divided, both the segments remain in perfect contact with their natural connexions, as completely nourished as before the operation; and, if the hæmorrhage occurs amid all these favourable circumstances, it forms the strongest argument against those who imagine the disturbance of the artery to be its cause." We should draw the contrary inference. How two ligatures and intermediate division of the artery can occasion less disturbance than one, we do not clearly comprehend. We should imagine that it creates more.

"Another cause that has been adduced as leading to secondary hæmorrhage is, the circumstance of the ligature having been applied near to a collateral branch, because, in such case, there can be no internal coagulum formed, and a part of the natural process must be incomplete. It has been already stated, that more importance seemed to have been attached to this early coagulum than it deserved; and, indeed, I cannot see how it can have any influence on an artery at so late a period as when the ligature is about to separate; but the best observation on this subject may be derived from experience. I have tied the common carotid, within an eighth of an inch of its origin from the innominata, with the most complete success: and yet, if there is one spot in the body where this cause of secondary hæmorrhage should operate with decided effect, it is here. This theory must, therefore, be dismissed as insufficient also." 187.

We apprehend that most unprejudiced surgeons will pronounce this hasty reasoning. A general rule, founded upon many facts, is tried by a single exceptional occurrence. As a matter of reasoning, the internal clot must be of service—as a matter of fact it has been found so, for the operation for aneurism has generally been most successful, on arteries with few collateral branches. If we might hint a fault in Mr. Porter, it is that he is too fond of arguing from exceptions.

Mr. Porter observes in continuation, that it seems to be nearly proved that, in a divided artery, a different process is set up in the two segments as preparatory to secondary hæmorrhage—that the superior one inflames *healthily*, lymph is effused, and the orifice obliterated—and that the inferior inflames *unhealthily*, lymph is not effused, the vessel remains open, and allows of the escape of the blood; the next, and the really valuable point to determine, is as to the cause which gives a tendency to this unhealthy inflammation in one individual more than in another.

Mr. Porter observes, that however hopeless the case may appear, when considered pathologically, it is certain that some patients have recovered, and, therefore, it is our duty to use our best exertions: and he thinks the case ought to be treated in the same manner, and on the same principles, as ordinary hæmorrhage, namely, the vessel must be subjected to the degree of pressure which would in such case cause its obliteration. Here the ligature is inadmissible, because, in the confusion of parts, it would be difficult, if not impossible, to find the artery, but more particularly, because that line of practice had already been tried and failed. There appears then to be no resource but the application of mechanical pressure by some other method, and if this does not accomplish the desired purpose, and the limb cannot be removed, the patient's chance of life is but small indeed.

Mr. Porter discusses the propriety of applying a fresh ligature on the artery nearer the heart. He inquires—"if the superior segment is closed and blocked up with lymph, or if, as I have before noticed, the ligature still remains upon it, whilst the lower had separated and was bleeding, of what possible utility could the tying of the artery higher up prove? It could only remove the impulse of the heart from the superior section, from which no danger is to be apprehended, but could have no influence on the lower, already deprived of that impulse, and supplied only by the collateral branches." Mr. Porter thinks the operation positively injurious in two ways; one by a fresh secondary hæmorrhage from the newly-tied artery, and the other, by inducing gangrene.

He adds:—

"On a prima facie view of the subject, it would be only natural to suppose that where the ligature of the femoral artery is followed by secondary hæmorrhage, the same causes operating in the same individual would occasion the same result if the iliac was secured afterwards. Such is really the case, but with this difference, that the hæmorrhage from the second ligature always appears at an earlier period than that from the first. Some years since, I had an opportunity of seeing this fact fatally exemplified, in a man who was the subject of operation for popliteal aneurism. In this case no ligature was used; but the artery, having been laid bare, was compressed with Deschamp's instrument; yet, in due course of time, secondary hæmorrhage made its appearance—the artery bled on the eleventh day. There was space for securing the vessel higher up on the thigh, which was accordingly done; the ligature here separated, and the bleeding occurred on the sixth day, the external iliac was then tied, but it bled on the third day. As a last resource, the thigh was amputated, although I never could learn what benefit was expected from this operation, and the patient died upon the table. In these remarks, relative to the second ligature of arteries, I am again borne out by the high authority of Dupuytren, who observes, that in these cases the cellular tissue of the artery becomes inflamed and hard, that it breaks down under the ligature, and, being thus incapable of retaining it, allows of its premature separation." 192.

If the second ligature is not followed by hæmorrhage, the patient is still, by no means safe, gangrene not unfrequently occurring. We think that the opinions of Mr. Porter on this subject deserve the consideration of surgeons, although we are not prepared to say that we go their entire length. Whatever theory may inculcate, there are still a sufficient number of cases of success from applying a second or even a third ligature to make us hesitate to sweep the operation from amongst the *possibilia* of surgery.

The treatment of the case which Mr. Porter recommends is this :—on the appearance of the blood the wound should be opened up, the granulations broken, and the mouth of the bleeding vessel fairly exposed; on it a small piece of prepared sponge should be placed deep in the bottom of the wound, care being taken that the pressure should be confined to this one spot—on this a small compress of lint, and another, and another, successively, until the graduated compress shall have attained a level higher than the adjacent surface of the limb. This must be firmly retained without being permitted to slip, and without allowing the escape of a single drop of blood; and, if this can be accomplished for three or four days, there is every reasonable probability of success. But here is the great difficulty. It is scarcely possible to apply a bandage, so as to answer the purpose, without interfering with the venous circulation and occasioning great pain; and as all bandages stretch and slip, and become relaxed, no certain reliance can be placed on them in a case where the patient's feelings encourage him to disturb them.

Now, let us suppose this pressure applied and maintained in the best possible manner—suppose a number of assistants relieving each other in keeping up a continued manual pressure, and regulating both the quantity and direction of it to the extent that might be necessary, and no more, still is the case unpromising: for the artery is seldom in a condition to effect those changes necessary to the permanent suppression of the bleeding, and although some few recover, yet is the chance against any given case exceeding great. For the management of one of these cases Sir P. Crampton contrived a kind of tourniquet, which has hitherto proved useful, and, probably, will be successful where success is possible. It consists of a plate on which the back of the thigh rests, to which is attached an iron hoop-ring; in the front of this ring a screw is placed, which, acting on a plate underneath, makes direct pressure on the graduated compress, whilst the hoop protects every other part of the circumference of the limb. By means of this instrument two cases were treated in the Meath Hospital with the most remarkable success, and some others, Mr. Porter believes, in Dr. Stevens'; it may be removed if the bleeding continues subdued for a few days; but the sponge at the bottom of the wound should be left to be detached by suppuration. But in all the cases of recovery, abscesses formed in the superior and external parts of the thigh, pending the pressure or immediately after its removal; in one of them the wound became sloughy, and fears were entertained that it would be necessary to discontinue the pressure. The first on whom it was tried seemed to recover well, but, in a short time afterwards, was attacked with abscesses, disease of the knee-joint, periostitis, and finally the limb was amputated for spreading gangrene. Mr. Porter has seen two other cases treated by compression end fatally.

Sometimes the patient complains of intolerable pain from the pressure, the limb becomes œdematous, and gangrene supervenes—sometimes the part of the thigh immediately around the wound is seized with erysipelas, which spreads in every direction, notwithstanding the removal of the pressure—sometimes the edges of the wound become livid, dark purple, or black, and gangrene commences there. In all and every of these cases, the constitution is deeply and dangerously engaged. No case of secondary hæmorrhage can occur without great anxiety and excitement of mind, accompanied by the debility which is the natural consequence of repeated losses of blood. This combination produces an irritative fever of the worst kind, exhibiting symptoms of nearly typhoid depression: a system thus deranged is very unlikely to make any successful rally.

Such is the picture drawn of compression by its advocate. We must be excused if we adhere to the opinion we expressed, and believe that a second ligature may sometimes be judiciously employed. For many of the evils described by Mr. Porter are attributable to compression itself.

"There is still," says Mr. Porter, "another form of secondary hæmorrhage, more provoking in a surgical point of view, (if I may be allowed the expression,) than either of the former, because it comes on at a later period, after the ligature has been for some time separated and removed, and when both practitioner and patient might have entertained a reasonable expectation of having surmounted every danger. This, as far as I know, is a most calamitous occurrence, and scarcely admits either hope or chance of escape. Thus, when the wound remains a long time open and suppurating profusely, or when abscesses and sinuses form around the vessel, or when it lies bare and denuded at the bottom of the wound, there is danger that it will give way and slough, just as it might do in any gangrenous or phagedenic ulcer. It is probable that to this cause the failure of Mott's case of ligature of the innominata is to be attributed. The ligature came away on the fourteenth day after the operation, and on the twenty-third day the hæmorrhage occurred, bursting forth with great violence to the extent of twenty-four ounces, and nearly destroying the patient in a moment; he died on the twenty-sixth day, and dissection shewed a large and deep ulceration around the artery, which had spread to, and engaged it, as well as the other great vessels arising from the arch of the aorta.

Mr. Abernethy had a case nearly of this description in the femoral artery, and I have seen one in the arm, where the artery was completely insulated by a sinus running along it. Fortunately these cases are not of frequent occurrence, for they scarcely admit of any remedy except the deplorable one of amputation, and if the situation of the vessel will admit of this, I know not how life could be preserved; for it would be impossible to apply pressure on a vessel in the midst of such an open sore or abscess; and I have already shewn how very inefficacious a ligature must be when placed higher up or nearer to their heart. It is obvious that this kind of consecutive hæmorrhage must frequently owe its origin to constitutional causes, and also that the place in which the artery is situated may occasionally have some influence. Thus, as these protracted unhealthy suppurations are prone to occur where there is abundance of loose cellular texture, such as the anterior mediastinum or the axilla, perhaps there is less reason to calculate on the successful results of operations in such localities—at all events they are not the situations that would be chosen if there existed a power of selection." 199.

*Aneurismal Varyx and Varicose Aneurism.*—After noticing the occurrence and nature of these forms of aneurismal disease, Mr. Porter remarks, that perhaps there may be some fortuitous circumstances, connected with the size or the direction of a wound that may dispose to this particular result, and with which we are unacquainted, and thus the communication may be established between vessels in a normal and healthy state. But he thinks there is something in the vessels themselves, which leads him to notice a condition of artery and vein that, if it does not occasionally produce this affection, seems strongly to predispose to its occurrence. Every anatomist is acquainted with the close adhesion that exists between the popliteal artery and vein in the adult male, an adhesion so frequent and so firm that it may almost be regarded as the natural condition of these vessels in such subjects; now, it must be quite obvious that, in the event of a perforating wound, such previously formed attachment will greatly facilitate the establishment of an aneurismal communication; and Mr. Porter has seen such attachment between the vessels in other situations, probably the result of chronic inflammation, often, for instance, in the thigh. Mr. Porter is not aware that this condition has been noticed by Scarpa, or other writers who have described the disease, they, generally, regarding it only as the result of injury, and so explaining its accidental formation between healthy vessels; but, let us admit for a moment the possibility of its being idiopathic, and such explanation must be evidently imperfect. On the other hand, in the event of ulceration attacking an artery in a spot corresponding with an adherent vein, it is not at all impossible but that a communication may thus be formed. Mr. Porter, indeed, has never seen a fusiform dilatation of an artery, the very state in which ulceration is likely to occur, that the accompanying vein was not firmly adherent to it. In the Winter of 1839-40, a case of subclavian aneurism occurred in the Meath Hospital, in which the patient died suddenly, previous to the performance of any operation; and, on examination, two separate and distinct fusiform dilations were found, both of which were so strongly adherent to the vein as to be separated with difficulty without laceration. And, more recently still, a case of mixed aneurism of the femoral artery was examined in the same hospital, in which the vein was so adherent to the dilated artery as to compress it along the entire line of attachment, and cause the expanded portion to assume an hourglass shape.

With respect to the treatment of the affection, Mr. Porter is disposed to do little in the way of operative interference.

"If," says our author, "we shall be satisfied with preventing an increase of the tumour, very moderate compression will be sufficient; but this treatment is purely palliative, and immediately on the compression being discontinued, the varix will commence to grow again. If our aim is *permanently* to check the disease, the abnormal current of blood must be stopped, and this can only be effected by the obliteration, either of the artery or of the vein, or of both, by inflammation. Now, in seeking either of these objects, a good deal of discrimination is necessary; and I believe it will be important to modify our views according to circumstances. In the simple aneurismal varix I have already said that the mildest treatment will be the best, and I should be satisfied with such a degree of compression as would merely control the disease; but in this, as in every other form of aneurismal affection, there are circumstances that may forbid the employment of pressure at all. The varix may be so situated that compression could not be applied—in the neck, for instance, or in the axilla; or, being applied, it may occasion a degree of torture that cannot be endured; or it may produce œdema or numbness of the limb—in short, the same objections may be raised to it that have been applied to compression generally.

Dupuytren is very decided in his reprobation of pressure; he says it is very difficult to employ, very inconvenient in general, and, according to the seat of the affection, occasions dangerous swelling and intolerable pains: moreover, it is inefficacious in obliterating an old aperture, rounded off, and organized like the lining membrane of arteries and veins, circumstances in which the ligature itself almost always fails. Doubtless these observations are perfectly true—there are cases in which compression cannot be tried, and others in which it cannot be borne, but they are very few, and in considering this point, and in testing it practically hereafter, let us attach a definite meaning to the term. Dupuytren, and the other opposers of compression, speak of it as a *curative* means—I, only as a *palliative:* they seek to stop the abnormal current of blood—I, only to moderate its force, and it is probable both parties are right. I know that, in some fortunate cases, compression has cured the disease; but, nevertheless, I should not be disposed to place much confidence in it, and would never resort to it whilst I possessed the alternative of performing an operation less tedious and less painful." 211.

An operation is more likely to be requisite for varicose aneurism than for aneurismal varyx. The aperture from the sac into the vein may be less free than that into the artery, and hence the sac may increase. What operation is to be preferred? Mr. Porter observes that, according to Dupuytren, Hunter's operation always fails on account of the numerous anastomoses carrying the blood round to the seat of the aneurism; and the observation is a correct one, though not sufficiently explicit. The varicose aneurism is, as to the principle on which a cure is to be effected, precisely in a similar condition to the traumatic aneurism—that is, there is an aperture in the sac through which fluid blood can escape—the sac, therefore, can never be filled by a coagulum, or brought so to press upon the injured artery as to cause its obliteration. The Hunterian operation, then, will be a palliative, as long as it removes the impulse of the heart from the tumour; but the moment that impulse is re-conveyed, by the collateral circulation, it becomes completely useless. It may, therefore, be performed, and may appear to be successful in particular situations, but it is wholly inapplicable where an extensive collateral circulation exists —as, for instance, in the internal carotid. There only remains then the operation of cutting down on the artery, and tying it above and below the wound; an operation often exceedingly difficult. For, first, if it be possible, the vein should be spared—it ought not to be cut into, or tied, or otherwise interfered with; and it is often no easy matter to avoid it; for we know it is distended and swollen, lying in general immediately in front of the artery, so that the operator must go round it, if he wishes to reach the latter vessel in safety. Another cause of difficulty is the hæmorrhage; it is always a bloody operation when the tumour has reached to any considerable size, and happens to be opened; and, although the bleeding may not be such as to bring life into absolute peril, yet it always obscures the parts; and it is particularly difficult to obviate this source of inconvenience; for the same pressure that will control the flow of blood from the artery will encourage and increase that from the vein. These reasons will be quite sufficient to teach any operator the utmost caution in meddling with the vein. Suppose, now, that the surgeon has been able to expose the artery, he finds it almost constantly in an abnormal or unhealthy condition—very often it is dilated—always it is so firmly adherent to the vein, for an inch or more above and below the communicating aperture, that it cannot be separated by dissection, and the vein is greatly exposed to injury from the passage of the needle. Lastly, this very condition of the artery predisposes to secondary hæmorrhage; and it is a practical fact that this calamitous occurrence very often happens after this operation.

This concludes our account of the work before us. The length of that account is an evidence of the favourable opinion we entertain of Mr. Porter's observations. There is much originality in many of them, and all possess a practical value. And if we conceive that Mr. Porter is apt to look too earnestly upon exceptions, and over-rate their importance, we may possibly do him an injustice.

